# *Drosophila* Adducin facilitates phase separation and function of a conserved spindle orientation complex

**DOI:** 10.3389/fcell.2023.1220529

**Published:** 2023-08-16

**Authors:** Amalia S. Parra, Cameron A. Moezzi, Christopher A. Johnston

**Affiliations:** Department of Biology, University of New Mexico, Albuquerque, NM, United States

**Keywords:** neural stem cell, asymmetric cell division, spindle orientation, phase separation, cell polarity

## Abstract

Asymmetric cell division (ACD) allows stem cells to generate differentiating progeny while simultaneously maintaining their own pluripotent state. ACD involves coupling mitotic spindle orientation with cortical polarity cues to direct unequal segregation of cell fate determinants. In *Drosophila* neural stem cells (neuroblasts; NBs), spindles orient along an apical-basal polarity axis through a conserved complex of Partner of Inscuteable (Pins; human LGN) and Mushroom body defect (Mud; human NuMA). While many details of its function are well known, the molecular mechanics that drive assembly of the cortical Pins/Mud complex remain unclear, particularly with respect to the mutually exclusive Pins complex formed with the apical scaffold protein Inscuteable (Insc). Here we identify Hu li tai shao (Hts; human Adducin) as a direct Mud-binding protein, using an aldolase fold within its head domain (Hts^HEAD^) to bind a short Mud coiled-coil domain (Mud^CC^) that is adjacent to the Pins-binding domain (Mud^PBD^). Hts is expressed throughout the larval central brain and apically polarizes in mitotic NBs where it is required for Mud-dependent spindle orientation. *In vitro* analyses reveal that Pins undergoes liquid-liquid phase separation with Mud, but not with Insc, suggesting a potential molecular basis for differential assembly mechanics between these two competing apical protein complexes. Furthermore, we find that Hts binds an intact Pins/Mud complex, reduces the concentration threshold for its phase separation, and alters the liquid-like property of the resulting phase separated droplets. Domain mapping and mutational analyses implicate critical roles for both multivalent interactions (via Mud^CC^ oligomerization) and protein disorder (via an intrinsically disordered region in Hts; Hts^IDR^) in phase separation of the Hts/Mud/Pins complex. Our study identifies a new component of the spindle positioning machinery in NBs and suggests that phase separation of specific protein complexes might regulate ordered assembly within the apical domain to ensure proper signaling output.

## 1 Introduction

Stem cells generate diverse differentiated cell types throughout development but must balance this against their own self renewal to maintain an adequate stem cell pool. Disrupting this balance has been associated with developmental defects as well as cancer (Sunchu and Cabernard, 2020), underscoring the importance of defining the molecular mechanisms of this conserved process. Distinct cell fate acquisition is achieved through asymmetric cell division (ACD), which generates two molecularly non-identical progeny cells (e.g., one self-renewing stem cell and one differentiating progenitor cell). Across diverse stem cell types, ACD is orchestrated through two intersecting processes, cortical polarity and mitotic spindle orientation, the core components of which have been shown to be evolutionarily conserved (Dewey et al., 2015a). Neural stems cells (neuroblasts; NBs) in the developing *Drosophila* central nervous system are a well-studied and proven model system for studying the molecular mechanisms underpinning these core ACD events (Homem and Knoblich, 2012). These neural stem cells are responsible for generating the broad cell type diversity within the central nervous system and have provided valuable insight into the role of ACD in neurogenesis (Doe, 2017).

In the early phases of mitosis, NBs establish an apical-basal polarity axis through the function of the apical Par complex (Par3/Bazooka [Baz], Par6, and aPKC). Subsequently, the mitotic spindle aligns to this polarity axis via the apically polarized Partner of Inscuteable (Pins) complex (Schaefer et al., 2000; Schaefer et al., 2001). Pins directs spindle orientation through direct interactions with two key effectors. First, Pins binds the tumor suppressor protein Discs large (Dlg) to capture microtubule plus-ends via the kinesin Khc-73 (Siegrist and Doe, 2005; Johnston et al., 2009). Second, Pins binds Mushroom body defect (Mud) to generate spindle forces via the Dynein motor complex (Bowman et al., 2006; Siller et al., 2006). The Pins/Mud complex has been particularly well-studied in diverse cell types across taxa (Dewey et al., 2015a; di Pietro et al., 2016), yet key knowledge gaps remain in our understanding of its molecular functions. Perhaps most notably, the ability of Pins to directly bind Mud is mutually exclusive with its interaction with Inscuteable (Insc), an adaptor protein that is also apically polarized in NBs (Culurgioni et al., 2011; Zhu et al., 2011; Mauser and Prehoda, 2012). Insc, which also binds Baz, was originally proposed to scaffold the apical polarity and spindle orientation complexes as a means of molecularly tethering these two core functions of ACD (Schober et al., 1999). This model was subsequently challenged by biochemical and structural studies demonstrating competitive, rather than complementary, Pins/Insc and Pins/Mud complexes (Mapelli and Gonzalez, 2012). Despite the competitive nature of these complexes, however, all three of these apical components are necessary for proper spindle positioning. How NBs, and potentially other cell types requiring the activity of these complexes, resolve this apparent paradox remains unanswered, and refined molecular models adequate to explain their mutual functions have not been proposed.

**FIGURE 1:**
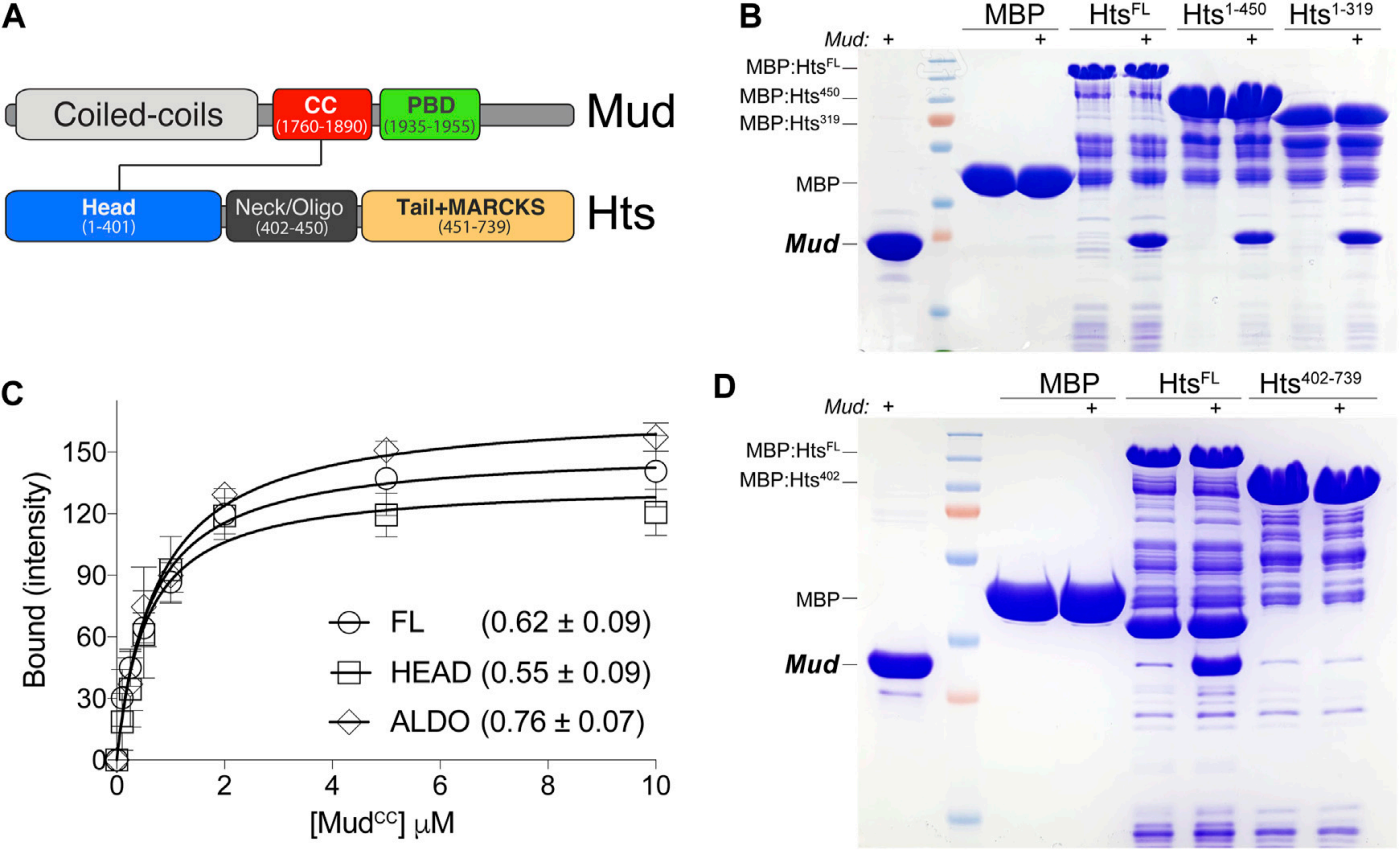
Hts directly binds Mud *in vitro*. **(A)** Domain architectures of Mud (*top*) and Hts (*bottom*). Mud contains extended coiled-coils at the N-terminus (CC; *light grey*), followed by the critical Mud^CC^ domain (*red*) and Pins-binding domain (PBD; *green*) studied herein. Hts contains an N-terminal “Head” domain with an imbedded aldolase_II fold (*blue*), a central oligomerization “Neck” domain (*dark grey*), and a C-terminal “Tail” domain that is capped with a Myristoylated Alanine Rich Protein Kinase C Substrate sequence (MARCKS; *orange*). Note that diagrams are not to scale with respect to overall protein lengths (Mud, 2,401 amino acids; Hts, 718 amino acids). **(B)** Representative gel (of 4 independent experiments) demonstrating binding of purified Mud^CC-PBD^ to the indicated Hts constructs, each as MBP fusions immobilized on amylose resin. Lane pairs show reactions in the absence and presence (indicated with “+”) of 5 μM Mud protein. Truncation of the Tail domain alone (Hts^1-450^) or together with the Neck (Hts^1-319^) did not reduce binding compared to full-length Hts protein (Hts^FL^). Negligible binding was detected with MBP alone as a control. **(C)** Saturation binding experiments reveal affinity of the Mud interaction with Hts. MBP-fused Hts (FL, full-length; HEAD, Head domain; ALDO, aldolase_II subregion of the Head domain) was immobilized on amylose resin and subsequently incubated in the absence or presence of soluble Mud^CC-PBD^ protein at the indicated concentrations. Curves shown were constructed using a one-site binding isotherm model, and the average ± standard deviation of the dissociation binding constants (K_D_) of 4 independent experiments are listed. **(D)** Representative gel (of 4 independent experiments) demonstrating binding of purified (6xHis)-Mud^CC-PBD^ to the indicated Hts constructs, each as MBP fusions immobilized on amylose resin. Lane pairs show reactions in the absence and presence (indicated with “+”) of 5 μM Mud protein. N-terminal truncation of the Head domain (Hts^402-739^) results in a loss of binding compared to full-length Hts protein (Hts^FL^). Negligible binding was detected with MBP alone as a control.

Mud is a large structural protein comprised of extended N-terminal coiled-coil domains followed by the minimal Pins-binding domain (Mud^PBD^) near the C-terminus (Figure 1A). Mud, as well as its human ortholog NuMA, localize to spindle poles to facilitate spindle assembly and maintain its bipolar structure (Radulescu and Cleveland, 2010; Bosveld et al., 2017). Mud recruitment and retention at the apical cell cortex is further necessary for its function in spindle orientation (Kotak et al., 2012; Fielmich et al., 2018; Okumura et al., 2018). We previously described a role for a short Mud coiled-coil domain (referred to herein as “Mud^CC^”; Figure 1A) that immediately precedes the Mud^PBD^ in regulating its interaction with Pins as well as specifically in its cortical localization (Dewey et al., 2015b). This Mud^CC^ binds intramolecularly with Mud^PBD^ to reduce the affinity of Pins binding, an effect that was reversed by Mud^CC^ phosphorylation by Warts kinase (Dewey et al., 2015b). Mud has numerous splice isoforms that are thought to contribute to specific functions (Taniguchi et al., 2014), and alternative splicing for inclusion or skipping of the Mud^PBD^-containing exon also affects Mud^CC^, with the NB isoform containing both domains (Siller et al., 2006). Taken together, these observations suggest that the Mud^CC-PBD^ domain tandem may function as a cohesive unit, prompting us to explore additional roles for the Mud^CC^ domain in Pins/Mud-dependent spindle orientation.

Using an unbiased proteomic approach, we have identified Hu li tai shao (Hts), the fly ortholog of human Adducin, as a direct binding partner of the Mud^CC^ domain. We find Hts is apically polarized in mitotic NBs. Although dispensable for NB polarity, Hts is required for Mud-dependent spindle orientation. Further, we find that the Pins/Mud complex, but not the Pins/Insc complex, undergoes liquid-liquid phase separation (LLPS) *in vitro*. Phase separation requires not only direct Pins/Mud binding but also formation of an intact, oligomeric Mud^CC^ coiled-coil. Hts directly associates with the Pins/Mud complex, lowers the critical concentration threshold for its LLPS, and appears to impact the liquid-like behavior of phase separated Pins/Mud droplets. Finally, we find that the Hts effects on Pins/Mud LLPS are dependent on a C-terminal intrinsically disorder region (Hts^IDR^). Our work identifies a new regulator of NB spindle orientation and suggests that phase separation may function as a molecular driver of complex organization within the apical polarity domain.

## 2 Results

### 2.1 Hts directly binds Mud *in vitro*

Mud is a large structural protein containing long, continuous coiled coil regions at the N-terminus, which are separated from the Pins-binding domain (Mud^PBD^) and a putative microtubule-interacting motif at the C-terminus (Figure 1A). Alternative splicing uniquely yields an additional, small coiled-coil domain adjacent to the Mud^PBD^ (referred to here as “Mud^CC^”) within the spindle orienting isoform expressed in NBs (Siller et al., 2006). We previously described a phosphorylation-sensitive intramolecular interaction between the Mud^CC^ and Mud^PBD^ domains that regulates its cortical localization and spindle orientation in wing disc epithelial cells (Dewey et al., 2015b), suggesting the tandem Mud^CC-PBD^ cassette may function as a concerted structural unit. Coiled-coils are regarded as multifaceted protein-protein interaction domains (Truebestein and Leonard, 2016), thus we performed mass spectrometry on samples isolated from pulldowns using recombinant GST:Mud^CC^ as bait and *Drosophila* S2 cell lysate as prey to identify new Mud^CC^-binding proteins that may contribute to such functions. Analysis in Scaffold4 software of these results yielded 25 unique proteins, among which was Hts [2 peptides at strict 99.9% protein and 95% peptide thresholds; also see (Cutillas and Johnston, 2021)]. Although neither a direct functional link nor physical interaction have been formally established previously between Hts and Mud, both proteins are known to localize to spectrosomes, cytoskeletal structures that play essential roles in the ACD of germline stem cells (Yu et al., 2006), suggesting the association identified here could have functional implications *in vivo*.

To corroborate the mass spectrometry findings, as well as probe if the interaction is direct, we next conducted equilibrium binding pulldown experiments using recombinant, purified proteins *in vitro*. Hts was fused to Maltose-binding protein (MBP) and coupled to amylose resin as the solid phase bait; Mud^CC-PBD^ was purified using a cleavable hexahistidine tag and used as the soluble prey component. As shown in Figure 1, Mud^CC-PBD^ bound to full-length Hts (Hts^FL^), with saturation binding isotherm experiments demonstrating a robust, sub-micromolar affinity. We next performed domain mapping experiments with Hts to identify the Mud binding site. The Hts N-terminal “Head” domain (Hts^HEAD^) contains a class II adolase fold (Hts^ALDO^) that is known to have lost enzymatic activity but remains otherwise poorly understood functionally (Matsuoka et al., 2000). This is followed by the “Neck” domain (Hts^NECK^) that contributes to oligomerization, with tetramers constituting the most likely functional unit. Finally, a C-terminal “Tail” domain (Hts^TAIL^), which includes a Myristoylated alanine-rich C-kinase substrate (MARCKS) region, binds F-actin and associates with additional components of the cortical actin cytoskeleton as well as other proteins (Matsuoka et al., 2000). Truncation of neither the Hts^TAIL^ alone nor in combination with the Hts^NECK^ impaired binding, implicating the Hts^HEAD^ as the primary site of Mud interaction (Figure 1B). In support of this inference, Mud binding affinity to the Hts^HEAD^ domain, as well as the isolated Hts^ALDO^ subdomain, was statistically indistinguishable from that of Hts^FL^ (Figure 1C). Finally, we examined Mud binding to an N-terminal truncation that deletes the Hts^HEAD^ domain, which showed a loss of binding compared to Hts^FL^ (Figure 1D). We conclude that Mud^CC^ directly binds the Hts^HEAD^ domain, specifically within the aldolase fold region.

### 2.2 Hts is expressed in the larval central brain and apically polarizes in mitotic NBs

**FIGURE 2:**
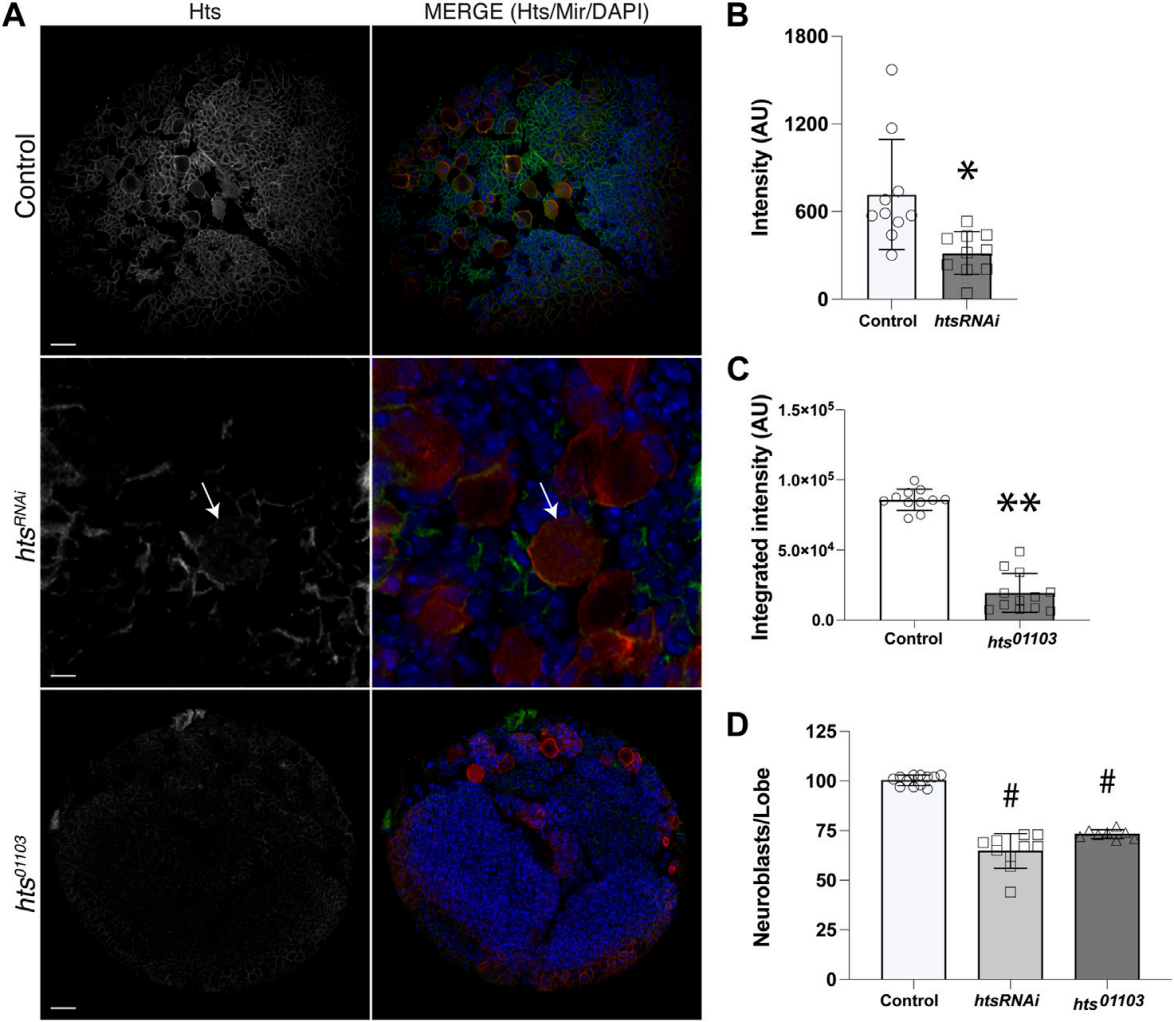
Hts is expressed in neuroblasts and throughout the larval brain. **(A)** Representative images of brains dissected from Control, *hts* ^*RNAi*^ (expressed specifically in NBs using a cell-specific driver), and *hts* ^*01103*^ third instar larvae and immunostained against Hts (*grey separated channel, left panels; green, right panels*) and Mir (*red, right panels*), along with DAPI (*blue, right panels*). Note that images for *hts* ^*RNAi*^ depict a selected region of the central brain to illustrate individual Mir^+^ NBs more clearly (e.g., NB marked with *white arrow*). Scale bar, 20 μm (*top, bottom*) and 5 μm (*middle*). **(B)** Quantification of Hts staining intensity in NBs of Control and following cell-specific expression of *hts* ^*RNAi*^. Hts knockdown results in a significant reduction of antibody labeling. *, *p* < 0.01 unpaired *t-test*. **(C)** Quantification of Hts staining intensity in central brain lobes of Control and *hts* ^*01103*^. Hts mutation results in a significant reduction of antibody labeling. **, *p* < 0.0001 unpaired *t-test*. **(D)** Quantification of NB cell counts, expressed as NBs per central brain lobe, in Control, *hts* ^*RNAi*^, and *hts* ^*01103*^. Both RNAi-mediated knockdown and allelic mutation of Hts result in significantly fewer NBs. ^#^, *p* < 0.0001 ANOVA with Tukey’s *post hoc* test.

Hts localizes to the spectrosome, a cytoskeletal structure that controls spindle anchoring and microtubule polarization in asymmetrically dividing germline stem cells (Lin et al., 1994; Robinson et al., 1994), yet to our knowledge a role in somatic stem cells such as neural stem cells has not been described. Confirmation of direct Mud binding thus prompted us to next investigate Hts expression and localization in central brain NBs. We first used an anti-Hts antibody to examine endogenous protein in third instar larval central brains (Zaccai and Lipshitz, 1996). This analysis revealed extensive Hts expression throughout brain lobes, including in Miranda (Mir)-positive NBs (Figure 2A). This result was similar to studies of Hts expression in the optic lobe at the same developmental stage (Ohler et al., 2011). Hts signal was significantly diminished throughout central brains dissected from the hypomorphic *hts* ^*01103*^ mutant [involving a P-element insertion that significantly reduces Hts protein expression (Ohler et al., 2011)]. Hts staining was also reduced in NBs expressing interfering RNA against *hts* (*hts* ^*RNAi*^) using the NB-specific *1407* ^*GAL4*^ driver (Figures 2A–C). Finally, to determine if Hts potentially plays a functional role in NBs, namely, in their ACD, we counted the number of Mir^+^ NBs in brain lobes among genotypes, as loss of polarity and/or spindle orientation genes often leads to abnormal NB numbers. Indeed, both mutant genotypes showed a reduction in NB population compared to control, which contained the stereotypical ∼100 central brain NBs per lobe (Figure 2D; Supplementary Figure S1). Hts function is explored further in the subsequent sections. Closer examination revealed that Hts was cortically localized in most cells, including NBs and their surrounding progeny, consistent with its association with cortical actin (Matsuoka et al., 2000). Unfortunately, however, this made assessing polarization of endogenous Hts localization in individual NBs challenging and ambiguous.

**FIGURE 3:**
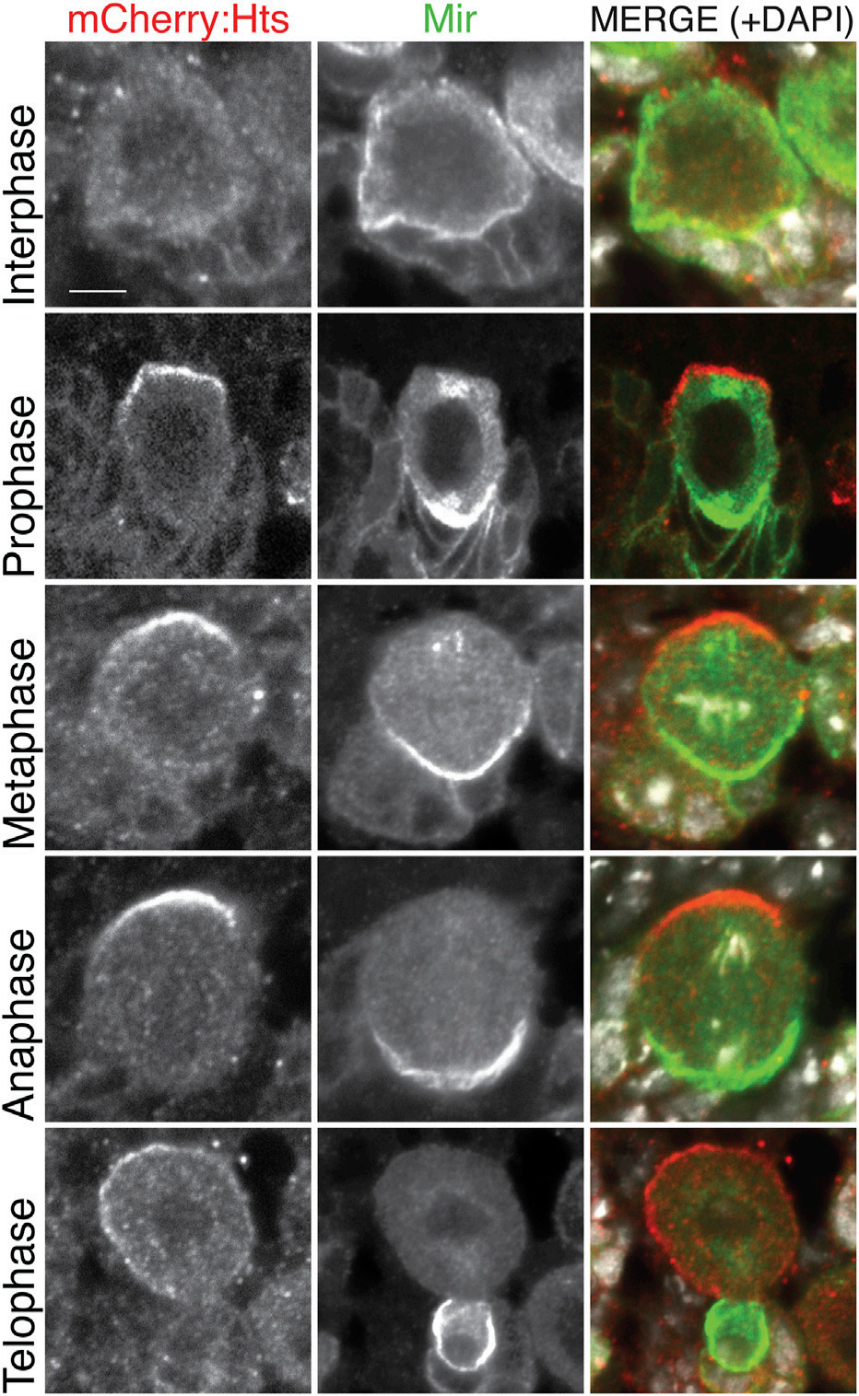
Hts is apically polarized in mitotic neuroblasts. The CNS was dissected from third instar larvae expressing mCherry-fused Hts (*red*; mCherry:Hts; note that the native mCherry fluorescence was used to visualize Hts) using the NB-specific *1407* ^*GAL4*^ driver and stained for Mir (*green*) along with DAPI (*white*). Individual NBs are shown to represent distinct cell cycle phases. mCherry:Hts is cytoplasmic during interphase and becomes apically polarized prior to nuclear envelope breakdown (i.e., “Prophase”), where it remains throughout mitosis. Telophase cells illustrate that Hts is asymmetrically segregated into the self-renewing NB. Cell cycle stages were inferred from a combination of Mir polarity (note that Mir is not polarized during interphase), the integrity of the nuclear envelope, and the pattern of DNA/DAPI organization. Scale bar, 5 μm.

To circumvent the ubiquitous antibody labeling of Hts, we next expressed a transgenic UAS:mCherry-Hts fusion, again under the control of NB-specific *1407* ^*GAL4*^ driver and examined localization of the native mCherry fluorophore. Notably, mCherry-Hts was apically polarized in mitotic NBs, opposite of the basal Mir crescent (Figure 3). Further inspection of NBs throughout the cell cycle revealed that Hts is nonpolarized with additional cytoplasmic localization during interphase, subsequently becoming polarized early in mitosis (e.g., prophase prior to nuclear envelope breakdown) where it remains through metaphase and anaphase (Figure 3). At telophase, Hts remained apical, segregating primarily into the nascent self-renewing NB progeny cell. At this stage, however, Hts also appeared to begin delocalizing back into the cytoplasm. Interestingly, this cell cycle-associated pattern of Hts localization closely mirrors that of aPKC (Oon and Prehoda, 2019), suggesting a potential functional link with the core apical polarity complex. We conclude that Hts is highly expressed in the larval central brain and a component of the apical polarity domain in mitotic NBs.

### 2.3 Hts is dispensable for apical-basal polarity in NBs

**FIGURE 4:**
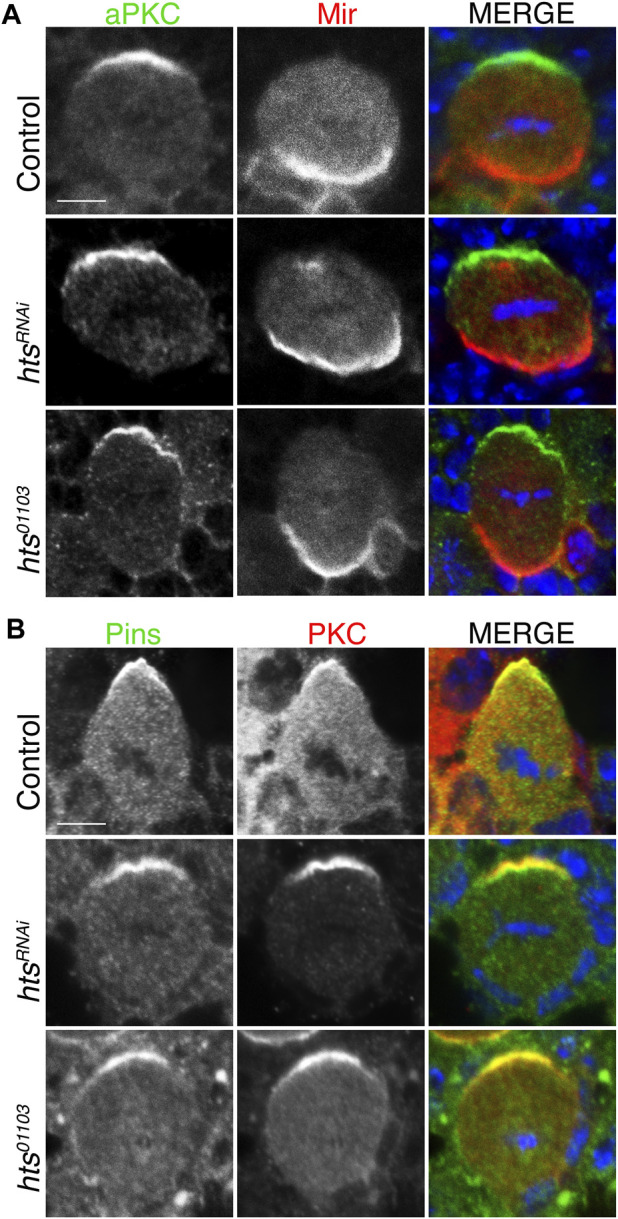
Hts is not required for apical-basal polarity in mitotic neuroblasts. **(A)** Brains were dissected from third instar larvae of indicated genotype and stained with antibodies against aPKC (*green*), Mir (*red*), and DAPI (*blue*). Loss-of-function Hts, through NB-specific RNAi expression or hypomorphic mutant allele, does not interfere with apical aPKC or basal Mir localization. Scale bar, 5 μm. **(B)** Brains were dissected from third instar larvae of indicated genotype and stained with antibodies against Pins (*green*), PKC (*red*), and DAPI (*blue*). As with aPKC, loss of Hts function does not interfere with apical Pins localization. Scale bar, 5 μm.

Having established Hts expression in the larval central brain and ability to apically polarize in dividing NBs, we next examined whether it contributes to localization of the core apical-basal polarity components. We found that NB-specific expression of *hts* ^*RNAi*^ did not alter apical localization of aPKC nor basal localization of Mir (Figure 4A). Similar results were obtained using the *hts* ^*01103*^ mutant allele. We further found that Pins also formed normal apical crescents following loss of Hts (Figure 4B). Overall, we conclude that Hts does not contribute to the establishment or maintenance of the core Par-mediated apical-basal polarity in NBs.

### 2.4 Hts is required for spindle orientation in NBs

**FIGURE 5:**
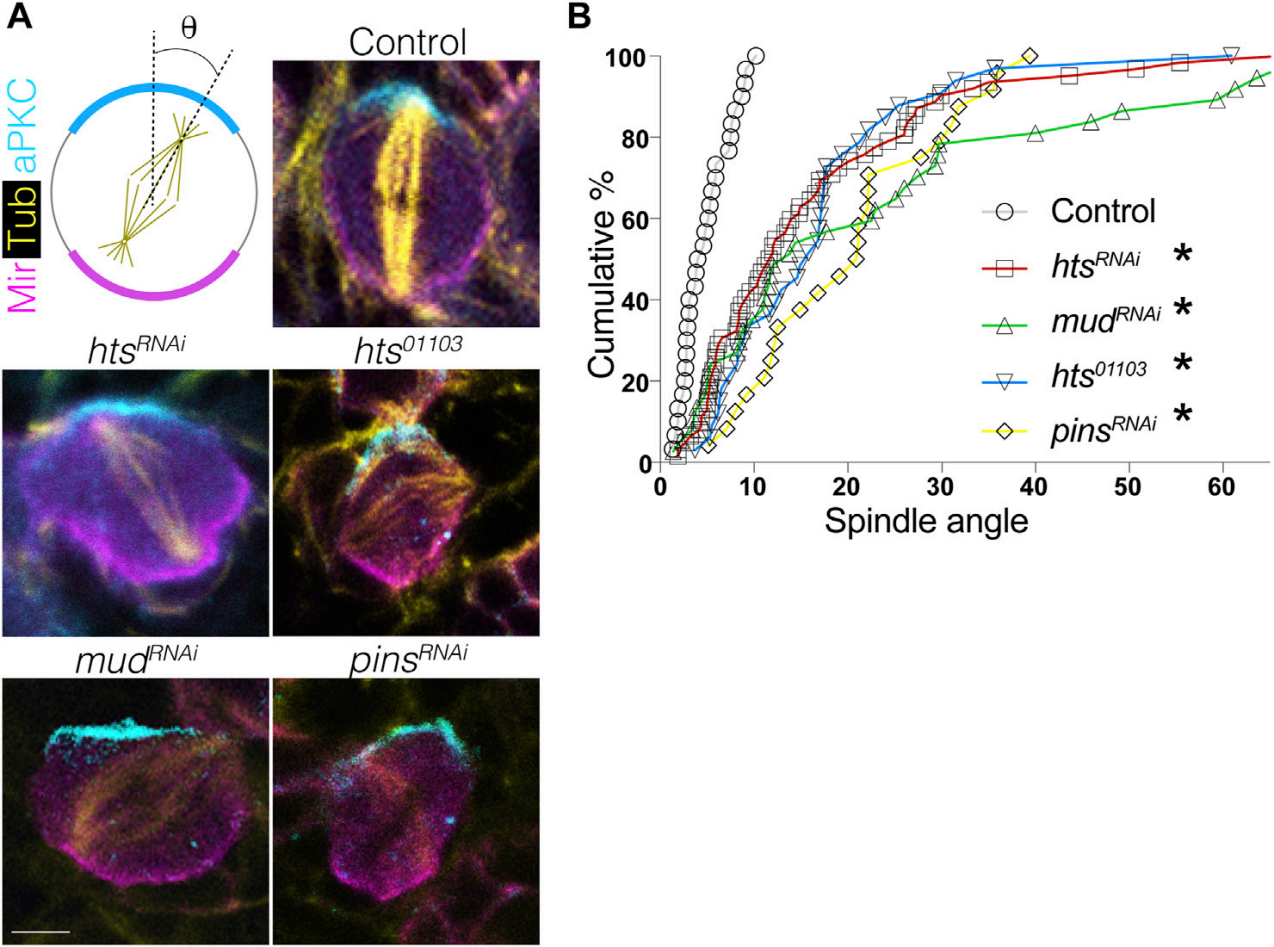
Hts is required for proper spindle positioning in mitotic neuroblasts. **(A)** Brains were dissected from third instar larvae of indicated genotype and stained with antibodies against aPKC (*cyan*), Mir (*magenta*), and α-tubulin to mark the spindle (*yellow*). Scale bar, 5 μm. **(B)** Spindle angles were measured for individual NBs using the center of the apical aPKC crescent as a reference (with 0° and 90° in either direction representing perfectly aligned and misaligned, respectively). Plots represent the cumulative percentage of NBs at or below the indicated spindle angle. Spindles in Control NBs (*grey circles*) accumulate within ∼10°, consistent with precision in spindle orientation, whereas *hts* ^*RNAi*^ (*red squares*), *mud* ^*RNAi*^ (*green triangles*), *pins* ^*RNAi*^ (*yellow diamonds*), and the *hts* ^*01103*^ loss-of-function allele (*blue inverted triangles*) each produce similar deficits. *, *p* < 0.01 ANOVA with Tukey’s *post hoc* test.

Having established that Hts directly binds Mud and is capable of apically polarizing in NBs, we next investigated its role in spindle positioning. Using the intact apical aPKC and basal Mir crescents as markers of the polarity axis, we found that expression of *hts* ^*RNAi*^ resulted in a spindle orientation defect similar to the expected impairment seen in response to *mud* ^*RNAi*^ as well as *pins* ^*RNAi*^ (Figure 5). Spindle angles were measured relative to the aPKC crescent center, with 0° representing ideal orientation and 90° indicating complete misalignment (Figure 5A). Plotting of spindle angles measured from numerous individual NBs as a cumulative percentage found a similar distribution between *hts, mud,* and *pins* knockdown, with each genotype showing spindle angles broadly distributed relative to control cells that cluster at lower, more precise angles of orientation (Figure 5B). Similar defects were also found in *hts* ^*01103*^. We conclude that Hts functions as a spindle positioning component likely through its interaction with Mud. However, that loss of Hts did not impact the localization of Pins the apical domain (Figure 4) suggested Hts could potentially impact Mud-dependent spindle orientation through a unique mechanism, which is explored further in the remaining sections.

### 2.5 Pins selectively phase separates when bound to Mud

**FIGURE 6:**
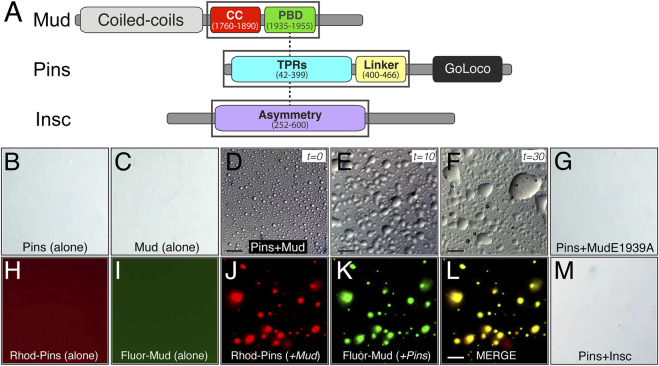
The Pins/Mud complex selectively phase separates *in vitro*. **(A)** Protein architectures of Mud (*top*), Pins (*middle*), and Insc (*bottom*) are shown with domains used in phase separation studies in black boxes. These include the coiled-coil (CC; *red*) plus Pins-binding domain (PBD; *green*) in Mud, the tetratricopeptide repeats (TPRs; *cyan*) plus Linker region (*yellow*) in Pins, and the Asymmetry domain (*purple*) in Insc. Dashed lines represent known direct, competitive interactions that Pins has with Mud and Insc (Zhu et al., 2011; Mapelli and Gonzalez, 2012; Mauser and Prehoda, 2012). **(B)** Pins (100 μM) incubated in LLPS buffer (20 mM Tris, pH 7.5 and 120 mM NaCl) alone does not form LLPS droplets. **(C)** Mud (100 μM) incubated in LLPS buffer alone also does not form LLPS droplets. **(D)** Pins (100 μM) and Mud (100 μM) incubated together in LLPS buffer readily form spherical LLPS droplets. Scale bar, 10 μm. **(E)** Pins/Mud LLPS droplets begin fusion events within 10 min (t = 10). Scale bar, 10 μm. **(F)** Pins/Mud LLPS continue to fuse over 30 min (t = 30) into large, wetted droplets. Scale bar, 10 μm. **(G)** Pins (100 μM) and an E1939A mutant Mud^PBD^ (100 μM) incubated together in LLPS buffer do not form LLPS droplets. **(H,I)** Rhodamine-labeled Pins (50 μM) or Fluorescein-labeled Mud (50 μM) were incubated alone in LLPS buffer. As with native proteins, neither dye-labeled protein formed LLPS droplets. **(J–L)** Rhodamine-labeled Pins (50 μM) and Fluorescein-labeled Mud (50 μM) incubated together in LLPS buffer form LLPS droplets that are positive for both rhodamine **(J)** and fluorescein **(K)** fluorescence, which is depicted in a merge image **(L)**. Scale bar, 10 μm. **(M)** Pins (100 μM) and Insc (100 μM) incubated together in LLPS buffer do not form LLPS droplets.

The required role for Hts in spindle positioning, despite a lack of effect on Pins polarity, prompted us to next explore more explicitly the mechanism for how Hts impacts function of the Pins/Mud complex. Considering its established role in organization of the actin cortex in other cell types (Matsuoka et al., 2000), we considered the possibility that Hts may influence the formation and behavior of the Pins/Mud complex within the intricate network of interactions comprising the apical polarity domain. Liquid-liquid phase separation (LLPS) has become an increasingly recognized mode of organizing supramolecular protein complexes, including those at the cell cortex, resulting in the formation of dense liquid-like droplets (Case et al., 2019). Furthermore, LLPS can facilitate inclusion of specific complex components at the exclusion of others within droplet phases, ultimately impacting their signaling outputs and cellular functions (Banjade and Rosen, 2014; Ditlev et al., 2018). We began by purifying recombinant Mud^CC-PBD^ and Pins^TPR−Linker^ protein domains and examining their ability to phase separate *in vitro* (Figure 6A). We found these proteins underwent LLPS when combined in a minimal biological buffer, forming numerous individual phase-separated droplets without the need for crowding agents such as PEG or Ficoll (Figure 6D). Isolated droplets underwent extensive fusion events over time consistent with liquid-like behavior (Ganser and Myong, 2020), eventually leading to formation of large wetted droplets (Figures 6E, F). Neither Mud nor Pins phase separated when examined alone, suggesting this phenomenon occurs specifically within the framework of an intact Pins/Mud complex (Figures 6B, C). Supportive of this inference was the fact that an E1939A missense mutation within the Mud^PBD^ that significantly impairs Pins binding was unable to phase separate with Pins under identical conditions [Figure 6G; (Zhu et al., 2011)]. To further corroborate the co-localization of Pins and Mud within observed LLPS droplets, we chemically labeled purified proteins with rhodamine and fluorescein, respectively, at N-terminal cysteine residues using maleimide-based fluorophores. Neither of these labeled proteins underwent LLPS alone (Figures 6H, I), however; when combined they phase separated under identical conditions to their native counterparts (Figures 6J–L). Importantly, the resulting droplets were uniformly positive for both fluorophore signals, providing strong evidence for this being a complex-specific and -driven process.

We next tested whether Pins can phase separate with its other, mutually-exclusive apical binding partner, the polarity adaptor protein Insc (Schober et al., 1999; Schaefer et al., 2000). In contrast to Mud, combining Pins with Insc did not lead to detectable LLPS droplet formation across a range of concentrations and buffer conditions tested with Mud (Figure 6M), spotlighting a specificity of this process with respect to Pins-binding partners. Failure to phase separate was not due to a lack of a direct interaction, as the purified Insc protein bound Pins with an affinity consistent with previous studies and well below the concentrations tested for LLPS [Supplementary Figure S2; (Zhu et al., 2011)]. We conclude that phase separation is likely a unique property of the Pins/Mud complex, with Pins/Insc instead remaining in a single unmixed phase. We suggest LLPS might therefore serve as a mechanism driving formation of discrete complexes within the apical polarity domain.

### 2.6 Mud^CC^ trimer formation is necessary for phase separation

**FIGURE 7:**
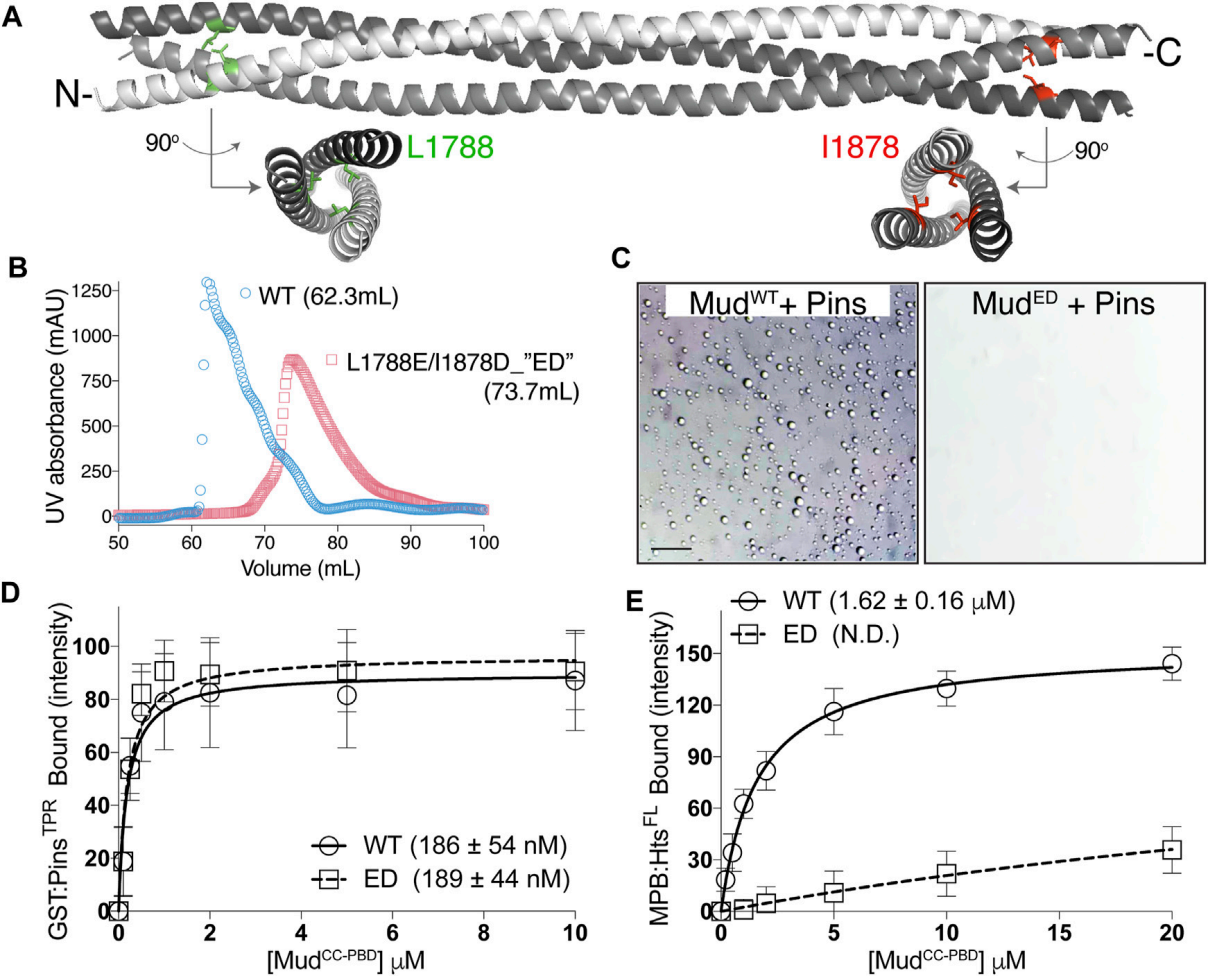
Oligomeric Mud^CC^ assembly is required for phase separation with Pins. **(A)** CCFold-generated homology model of Mud^CC^ depicts a parallel trimer (individual helices colored *white*, *grey*, and *dark grey*). Whereas the N- and C-terminal regions adopt an idealized coiled-coil structure, the central region deviates from this fold. Leucine-1788 (L1788; *green*) and Isoleucine-1878 (I1878; *red*) residues are depicted in stick mode along with rotated structures that demonstrate their “A-position,” core-facing orientations that likely contribute to stabilization of the coiled-coil structure. **(B)** Elution profiles of Mud^CC-PBD^ wild-type (WT; *blue circles*) and the L1788E/I1878D (ED) mutant (*red squares*) from a Sepharose-200 column with peak volumes indicated. The ED mutant elutes at a larger volume that is consistent with disruption of coiled-coil oligomerization. **(C)** Pins (100 μM) and Mud (100 μM), either WT or ED (*left* and *right*, respectively), were combined in LLPS buffer. Whereas Mud^WT^ readily forms LLPS droplets, the Mud^ED^ mutant fails to undergo phase separation. Scale bar, 10 μm. **(D)** GST-fused Pins^TPR^ domains were immobilized on glutathione resin and subsequently incubated in the absence or presence of the indicated concentrations of Mud^CC-PBD^, as either WT or ED mutant variant. Shown are saturation binding curves for combined results of 4 independent experiments, with the average ± standard deviation of the dissociation binding constant listed in parentheses. The ED mutation does not impair Pins binding. **(E)** MBP-fused Hts^FL^ was immobilized on amylose resin and subsequently incubated in the absence or presence of the indicated concentrations of Mud^CC-PBD^, as either WT or ED mutant variant. Shown are saturation binding curves for combined results of 4 independent experiments, with the average ± standard deviation of the dissociation binding constant listed in parentheses (N.D., not determined). In contrast to Pins in (E), the ED mutation significantly reduces binding to Hts.

Phase separation of heterotypic protein complexes is often driven by multivalent interactions, for example, those generated through repeated modular protein interaction domains in one or more components (Banjade and Rosen, 2014; Banani et al., 2017). Through their intrinsic oligomerization, coiled-coil domains necessarily generate a multivalent protein assembly, thus we next questioned whether an intact Mud^CC^ is required for phase separation with Pins. *In silico* primary sequence analysis predicted high coiled-coil confidence in Mud^CC^ apart from a short segment near the domain center, with a parallel trimer suggested as the most probably assembly. To explore this in further detail, we generated a structural model using CCFold, a threading-based algorithm specifically designed to build coiled-coil models from primary sequence input [Figure 7A; (Guzenko and Strelkov, 2018)]. This model was consistent with sequence analysis, revealing a parallel trimer of idealized coiled-coil structure, save for a short interruption near the domain center. We used this model to generate missense mutations at the N- and C-terminal ends predicted to disrupt coiled-coil formation. Specifically, amino acids were targeted at core-facing, hydrophobic “A” positions and mutated to electrostatic acidic residues (L1788E and I1878D; referred hereafter as “ED”). This Mud^ED^ mutant was highly expressed in *Escherichia coli* and stable to recombinant purification similar to wild-type protein; however, it eluted from size-exclusion column at a larger volume consistent with disruption of the coiled-coil trimer (i.e., a smaller molecular weight; Figure 7B).

We then examined the ability of the Mud^ED^ mutant to undergo phase separation when combined with Pins and found that it was completely devoid of this property compared to wild-type Mud under identical conditions (Figure 7C). Notably, however, Mud^ED^ fully retained its ability to bind Pins through its intact PBD (Figure 7D). These results demonstrate that Mud coiled-coil assembly is required for phase separation of the Pins/Mud complex, most likely through its creation of a multivalent interaction platform for Pins (e.g., a 3:1 stoichiometry assuming a trimeric Mud^CC^), and that the Mud^ED^ mutant decouples Pins binding from phase separation. Finally, we found that the Mud^ED^ mutant was significantly impaired in its ability to bind Hts (Figure 7E), consistent with the assembled Mud^CC^ being the site of this interaction. We conclude that although formation of an intact Mud^CC^ trimer is dispensable for Pins binding, it is required for phase separation as well as Hts binding.

### 2.7 Hts modulates Pins/Mud phase separation through a C-terminal IDR

**FIGURE 8:**
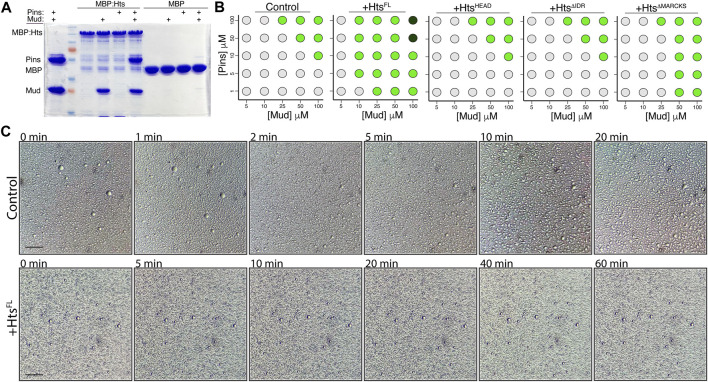
Hts binds the Pins/Mud complex and facilitates phase separation through its C-terminal intrinsically disordered region. **(A)** Representative gel (of 4 independent experiments) demonstrating binding of purified Mud^CC-PBD^, alone or in combination with Pins^TPR−Linker^, to MBP:Hts^FL^. In contrast, Pins was incapable of binding in the absence of co-incubated Mud. Minimal binding was detected with MBP. **(B)** Phase diagrams depict the concentration-dependence of droplet formation boundaries for the Pins/Mud complex alone (Control) or in the presence of the indicated Hts construct. Light grey circles represent no droplet formation, green circles represent formation of phase separated droplets, and dark green circles represent formation of dense precipitate without discernible droplets. Addition of Hts^FL^ reduces the concentrations of Pins and Mud necessary for droplet formation, whereas the Mud-binding Hts^HEAD^ domain is without effect. Truncation of the C-terminal intrinsically disordered region ( ${Hts}^{\Delta IDR}$ ) abolishes the effects of Hts, whereas selective removal of the MARCKS sequence ( ${Hts}^{\Delta MARCKS}$ ) reduces it. Scale bar, 10 μm. **(C)** Time course monitoring of Pins/Mud LLPS droplet dynamics in the absence (Control; *top*) or presence of Hts (+Hts^FL^; *bottom*). Time indications are listed in minutes post setup. Whereas droplets undergo extensive fusion events within 20 min in Control, those in the presence of Hts remain resistant to such dynamics for at least 60 min. Scale bar, 10 μm.

Having identified this distinct phase separation property of the Pins/Mud complex, we next explored how Hts, through its direct interaction with Mud, might interface with this process. We first tested the ability of Hts to bind an intact Pins/Mud complex, using the MBP:Hts^FL^ fusion with soluble Pins and Mud proteins. As shown in Figure 8A, Hts bound to Mud^CC-PBD^ but not Pins^TPR−Linker^ when each was examined alone. When combined, however, Hts was capable of pulling down both Mud and Pins, demonstrating that Hts, Mud, and Pins can mutually exist in a trimeric complex (Figure 8A). This is consistent with distinct binding sites on Mud for Hts and Pins (the CC and PBD regions, respectively). We conclude that Hts directly binds an intact Pins/Mud complex.

We next examined how addition of purified Hts^FL^ affected the concentration dependence of Pins/Mud phase separation. We found that addition of Hts significantly lowered the threshold concentrations of Pins and Mud necessary for droplet formation (Figure 8B). This is consistent with other recent findings that recruitment of specific “clients” can impact the phase boundary of otherwise autonomous phase-separating complexes (Banani et al., 2016; Banani et al., 2017). To understand the molecular aspects of this Hts-mediated effect, we then performed domain mapping experiments across identical conditions. Addition of the isolated Hts^HEAD^ domain, which is sufficient for Mud binding (Figure 1), did not impact the concentration regime under which Pins/Mud could phase separate (Figure 8B), revealing that Mud binding alone is insufficient for the effects of Hts and that additional sequence elements are necessary to influence LLPS. Inclusion of the Neck domain (e.g., Hts^HEAD−NECK^, also referred to as Hts^ΔIDR^) also did not affect phase separation, implicating the Hts C-terminal region as the region responsible for this property. *In silico* analysis of Hts sequence using PONDR yielded a high-confidence prediction of an intrinsically disordered region spanning the majority of the C-terminal and MARCKS domains [IDR; Supplementary Figure S3; (Romero et al., 2004; Uversky, 2020); http://www.pondr.com], consistent with previous analyses of human Adducin sequence (Gardner et al., 2013; Kruer et al., 2013). Interestingly, while removal of the entire C-terminal Hts^IDR^ completely abolished its effects on Pins/Mud LLPS, selective truncation of the Hts^MARCKS^ domain alone produced an intermediate loss-of-function (Figure 8B), suggesting this short sequence within the IDR imparts an important component of its effects. We conclude that Hts facilitates Pins/Mud phase separation by lowering the concentration-dependent phase boundary and that this effect is mediated through the Hts^IDR^.

To further explore how Hts might impact Pins/Mud phase separation dynamics, we examined droplet dynamics over time. Unlike those formed in the absence of Hts, which underwent liquid-like fusions within minutes of forming, phase separated droplets in the presence of Hts^FL^ were resistant to such events, remaining mostly as small individual droplets even after 1 hour of incubation (Figure 8C). The liquid property of LLPS droplets is known to have functional implications and has been suggested as means of preventing fusion among distinct phase separated complexes to maintain specificity (Boeynaems et al., 2018). The core apical polarity complex (e.g., Par3/Par6/aPKC) in NBs was recently shown to phase separate, and thus Hts-mediated alteration of Pins/Mud droplet dynamics may provide such control. We conclude that phase separated Hts/Mud/Pins complexes have altered biophysical properties compared to Pins/Mud.

**FIGURE 9:**
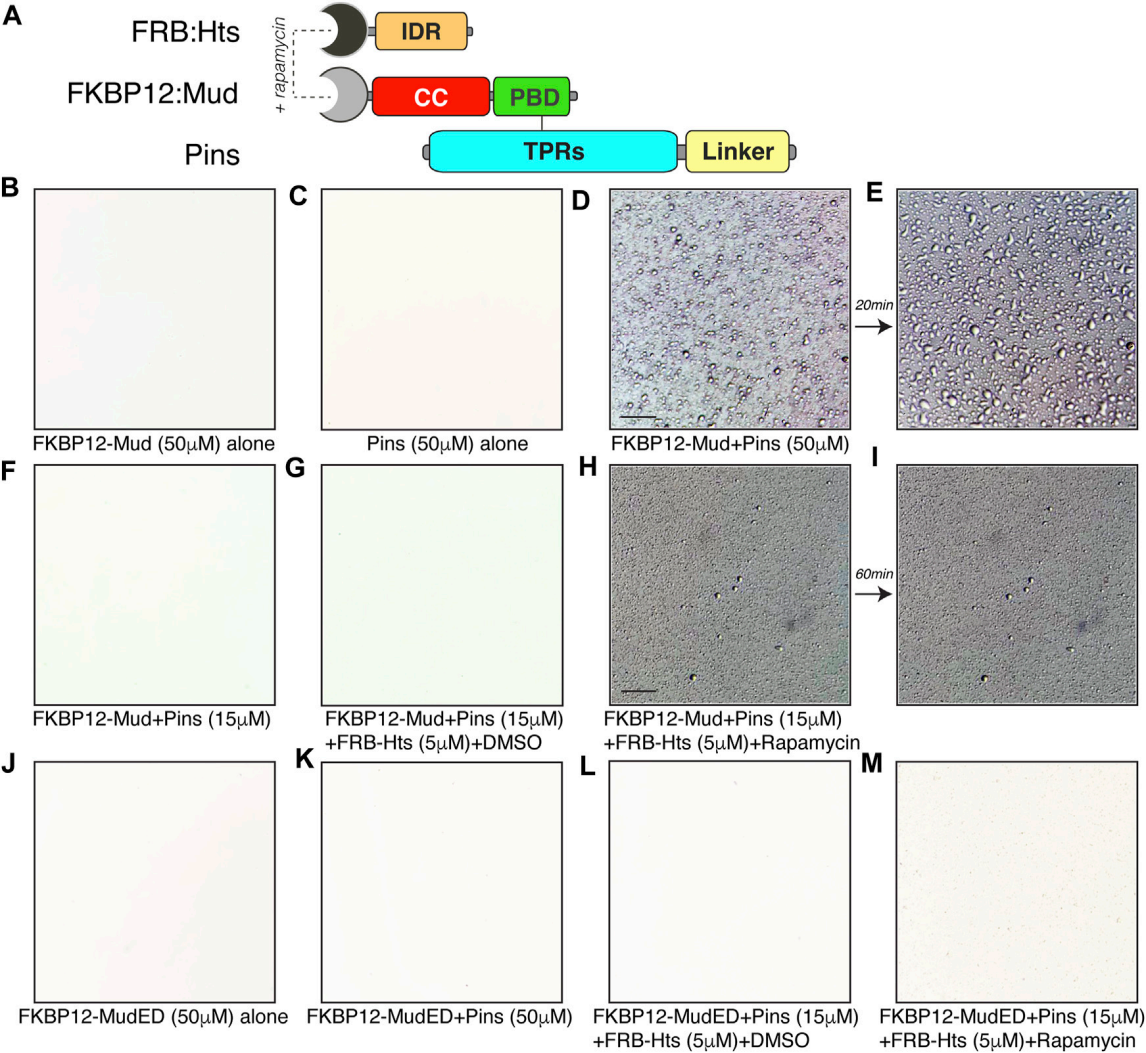
Hts^IDR^ is sufficient to induce Pins/Mud phase separation. **(A)** Domain diagrams depicting protein engineering of chemical-induced dimerization approach for fabricating an interaction between Mud^CC-PBD^ and the isolated Hts^IDR^. N-terminal fusions of FKBP-rapamycin binding domain (FRB) and the 12-kDa FK506 binding protein (FKBP12) were made to Hts and Mud, respectively. Dual-mode binding of rapamycin to these fusion domains can induce dimerization of the tagged Hts and Mud fragments despite their inability to bind natively. Untagged Pins^TPR−Linker^ protein was also used. **(B)** FKBP12-Mud (50 μM) alone in LLPS buffer did not produce phase separated droplets. **(C)** Pins (50 μM) alone in LLPS buffer did not produce phase separated droplets. **(D)** FKBP12-Mud and Pins (50 μM each) were combined in LLPS buffer and the resulting phase separated droplets were imaged. Scale bar, 10 μm. **(E)** Droplets formed in **(D)** underwent fusion events within 20 min **(F)** FKBP12-Mud and Pins (15 μM each) were combined in LLPS buffer and did not result in phase separation at these lower, sub-threshold concentrations. **(G)** Addition of FRB:Hts (5 μM) with DMSO solvent to identical conditions used in **(F)** did not result in phase separation. **(H)** Addition of FRB:Hts (5 μM) with rapamycin (1 μM) to identical conditions used in **(F)** resulted in formation of small phase separation droplets. Some precipitation was also noted, possibly caused by protein complex formation or rapamycin insolubility in LLPS buffer. Scale bar, 10 μm. **(I)** Small droplets formed in **(H)** failed to undergo fusion events within 60 min. **(J)** The “ED” Mud mutant FKBP12-Mud^ED^ (50 μM) alone in LLPS buffer did not produce phase separated droplets. **(K)** Mutant FKBP12-Mud^ED^ (50 μM) combined with Pins (50 μM each) in LLPS buffer did not produce phase separated droplets. **(L)** Addition of FRB:Hts (5 μM) with DMSO solvent to mutant FKBP12-Mud^ED^ (15 μM) combined with Pins (15 μM each) did not result in phase separation. **(M)** Addition of FRB:Hts (5 μM) with rapamycin (1 μM) to mutant FKBP12-Mud^ED^ (15 μM) combined with Pins (15 μM each) did not result in phase separation.

We next wondered if the Hts^IDR^ was not only required for its effects on LLPS but also sufficient. However, the Hts^IDR^ is dispensable for Mud binding, which occurs at the Hts^HEAD^ domain (Figure 1), precluding a direct examination of its effects. To circumvent this dilemma, we designed a rapamycin based chemical induced dimerization (CID) strategy to fabricate an interaction between Mud and the isolated Hts^IDR^ (DeRose et al., 2013). Specifically, we cloned and purified the human rapamycin-binding FKBP12 and FRB proteins as N-terminal fusions to Mud^CC-PBD^ and Hts^IDR^ domains, respectively (Figure 9A). Similar to native protein, FKBP12:Mud (nor Pins) did not phase separate when examined alone (Figures 9B, C). However, when combined with Pins at a high concentration (50 μM), FKBP12:Mud phase separated similarly to that observed with native proteins (Figure 9D), with droplets undergoing similar fusion events over time as well (Figure 9E). This indicates that FKBP12 fusion does not interfere with the normal LLPS dynamics of the Pins/Mud complex. At lower concentration (15 μM), no detectable LLPS was evident (Figure 9F), again consistent with non-tagged Mud protein seen previously. Addition of FRB:Hts^IDR^ in the absence of rapamycin (using DMSO as a control) did not trigger phase separation within this low concentration regime (Figure 9G). However, when FKBP12:Mud, Pins, and FRB:Hts^IDR^ were combined in the presence of rapamycin, small droplets were visible (Figure 9H). Notably, these droplets did not undergo fusion events over time (Figure 9I), similar to Pins/Mud droplets in the presence of Hts^FL^ (Figure 8C). We also tested the “ED” mutant form of Mud fused to FKBP12 as an additional control. This FKBP12:Mud^ED^ did not phase separate alone (Figure 9J), nor in combination with Pins (Figure 9K). Finally, addition of FRB:Hts^IDR^ and rapamycin was not capable of eliciting phase separation of FKBP:Mud^ED^ when combined with Pins (Figures 9L, M). Together, these results suggest that the Hts^IDR^, when physically bound with Mud, is sufficient to facilitate phase separation of the Pins/Mud complex.

## 3 Discussion

Mitotic spindle orientation plays a crucial role in both symmetric and asymmetric cell division across a range of tissue types in diverse model organisms, with many of the core molecular components being functionally conserved (Morin and Bellaiche, 2011; Lu and Johnston, 2013). Although not universal, the Pins/Mud complex appears to be an important regulator of spindle positioning in numerous cell types, both in stem and non-stem cells alike (di Pietro et al., 2016). One persistent knowledge gap regarding its molecular function, however, has been in understanding how the Pins/Mud complex remains competent in the face of a competing Pins/Insc complex within the apical NB domain (Mapelli and Gonzalez, 2012). LLPS has emerged as a means by which cells generate membraneless organizations of specific protein (or RNA) complexes within an otherwise intricate cellular milieu, including at the cell membrane (Ditlev et al., 2018; Parra and Johnston, 2022). Phase separation of these complexes can facilitate their function and increase efficiency. Indeed, the list of cellular functions suggested to be regulated by LLPS has seen continued growth in recent years (Lyon et al., 2021). We propose that selective phase separation of the Pins/Mud complex may organize its assembly within the NB apical domain to ensure efficient functional output and that Hts acts to promote this process.

Recent studies provide a contextual precedence for phase separation acting as a molecular driver of asymmetric cell division in NBs. For example, Numb and Partner of Numb (Pon), which form a basal localized complex critical to fate specification in the differentiating progeny cell, were recently found to phase separate *in vitro* (Shan et al., 2018). Similar to Pins/Mud, phase separation only occurred with the intact Numb/Pon complex generated from a multivalent assembly involving repeating interaction motifs within Pon. Moreover, disruption of such assembly impairs proper localization in NBs *in vivo* (Shan et al., 2018). Other studies demonstrated that the Par3/Par6 polarity complex also phase separates *in vitro*. In this case, Par3 was able to form droplets autonomously via oligomerization of its N-terminal region. Addition of Par6 lowered the concentration threshold for droplet formation (Liu et al., 2020), similar to the effects of Hts in our studies herein. Furthermore, aPKC can be recruited to phase separated droplets as a client protein and subsequently regulate their stability. Disruption of Par phase separation impaired NB polarity and cell fate specification, and Par3, Par6, and aPKC formed puncta, a common feature of phase separating proteins in cells, further suggesting a role for this process *in vivo* (Liu et al., 2020).

What then might be the purpose and advantage of a phase-separating spindle orientation complex? The de-mixed, two-phase system generated by LLPS can facilitate formation of distinct complexes—those included within phase-separated droplets and those remaining excluded in the dilute phase. Such selectivity can drive specific protein-RNA or -DNA complexes to control gene expression (Boija et al., 2018; Gibson et al., 2019; Guo et al., 2021), enhance kinetics of enzyme-catalyzed reactions (Peeples and Rosen, 2021; Zhang et al., 2021), and facilitate specificity and efficiency of membrane-delimited signal transduction (Case et al., 2019). Compartmentalization of specific protein complexes at the cell cortex is particularly relevant to the current studies. Although originally speculated to assemble as a large supramolecular complex, direct interaction between apical polarity and spindle orientation complexes has since been revealed as mutually exclusive. Specifically, the interaction of Pins with Mud, a requirement for spindle positioning, is mutually exclusive with Pins binding to the polarity scaffold protein Insc (Mapelli and Gonzalez, 2012). How this apparent paradox is resolved at the apical cortex has remained elusive. The ability of Pins to selectively phase separate when bound to Mud suggests this unique biophysical property of the Pins/Mud complex could underlie an organization within the apical domain that ensures productive spindle orientation output. Furthermore, the presence of Hts, which we find is also able to apically polarize in mitotic NBs, could augment this process by lowering the phase separation threshold and stabilizing the resulting droplets. These properties have been demonstrated in other IDR-containing proteins (Uversky, 2021), consistent with the requirement and sufficiency of the Hts^IDR^ impacting Pins/Mud phase separation. Emerging work on *C. elegans* P-granules suggests that intrinsically disordered proteins regulate the structural integrity of condensates by reducing droplet surface tension and impeding their coarsening (Folkmann et al., 2021). While it is tempting to speculate such a role for Hts, further experiments will be required to determine the exact impact of its IDR *in vivo*.

Another potential advantage of phase separation could be to enhance the signal output of the Pins/Mud spindle positioning complex. How cortical signaling complexes concentrate their components, organize and maintain their structures, and retain a regulated signaling efficiency within a crowded environment of other membrane-associated proteins and their surrounding cytoplasmic environment has begun to be explained by the inherent clustering of specific molecules by LLPS that can facilitate their function (Banani et al., 2017; Ditlev et al., 2018). Although direct evidence for such processes in spindle orientation complexes had not been described previously, our data is consistent with this model and is supported by other recent findings. For example, the human Mud ortholog, NuMA, was recently found to form discontinuous clusters at the cell cortex in HeLa cells (Okumura et al., 2018). Mutations that prevent formation of these clusters reduce spindle positioning accuracy despite not preventing cortical localization in general. These clusters were suggested to counteract the diffusion-prone nature of the microtubule force generating machinery at the cell cortex to enhance spindle rotations. Indeed, NuMA clustering was found to be critical to proper spindle force generation by the Dynein/Dynactin complex, suggesting it could function to organize multi-arm assemblies of this critical motor complex at the cortex similar to other locales such as the kinetochore (Dimitrova et al., 2016; Okumura et al., 2018; Urnavicius et al., 2018). Whether and how phase separation contributes to formations of cortical Mud/NuMA clustering will be important questions to resolve.

Based on our work herein, we propose that Hts acts to facilitate phase separation of the core spindle orientation complex at the polarized cell cortex in mitotic neural stem cells. Interestingly, numerous examples of membrane-associated complexes recently found to phase separate involve actin-binding factors (Su et al., 2021). Understanding how actin-associated Hts activity impacts Pins/Mud-dependent spindle positioning will be an exciting future endeavor. Many additional questions inform relevant future directions as well. Most fundamental will be to establish a *bone fide* relevance of this process *in vivo*, for example, through the introduction of LLPS-deficient Mud mutants. Determining if phase separated Pins/Mud complexes recruit Dlg, the complementary spindle orientation Pins effector (Johnston et al., 2009), would provide additional insight into how LLPS might facilitate organization at the apical NB cortex. It will also be interesting to determine if this process is conserved in mammalian and other systems (e.g., with human Adducin, LGN, and NuMA proteins). Finally, exploring a role for the Hts/Mud interaction in germline development, where both components have an established role in function of the spectrosome (Deng and Lin, 1997; Yu et al., 2006), could reveal additional insights that may be independent of LLPS (as Pins is not known to function with Mud in this context).

## 4 Materials and methods

### 4.1 *Drosophila melanogaster* husbandry and genetics

*Drosophila melanogaster* stocks were maintained on standard cornmeal medium at 20°C and crosses were raised at 29°C for all experiments unless otherwise noted.

### 4.2 Fly stocks

Driver Lines: 1407Inscuteable GAL4 (BDSC, #8751, RRID:BDSC_8751).

RNAi Lines: Hu Li Tai Shao (Hts) RNAi (BDSC, #38283, RRID:BDSC_38283).

Transgenic Lines: UAS-Hts:mCherry (BDSC, #66171, RRID:BDSC_66171).

Mutant alleles: hts^01103^ (BDSC, #10989, RRID:BDSC_10989).

### 4.3 Antibody staining

Whole brains from third instar larvae were dissected in cold (4°C) PBS followed by fixation for 23 min in 4% paraformaldehyde at room temperature. Tissues were washed three times at room temperature for 10 min in PBS-T (1x PBS, 0.3% Triton) and blocked for 1 h at room temperature using block buffer (PBS-T supplemented with goat and donkey serum 1x PBS, 0.3% Triton, 2.5% goat serum, 2.5% donkey serum) then incubated overnight in primary antibody solution diluted in block buffer at 4°C. Following incubation, tissues were washed three times for 20 min in block buffer followed by incubation in secondary antibody diluted in block buffer overnight at 4°C. Larval brains were mounted ventral side up in 80% glycerol and stored at 4°C until imaging.

The following antibodies were used: Rat Anti-Miranda (1:500) (Abcam, #197788, RRID:AB_2936368), Rabbit Anti-PKC (1:1,000) (Santa Cruz Biotechnology, #sc216, RRID:AB_23000359), Mouse Anti-Hts 1B1 (1:100, DSHB, RRID:AB_528289), Rat Anti-Partner of Inscuteable (Pins) (1:500, generous gift from Dr. Chris Doe; University of Oregon, United States, RRID:AB_2569570).

### 4.4 Image acquisition and processing

Images were acquired using a Zeiss LSM-780 confocal microscope equipped with a 63x or 40x oil immersion X 1.40 numerical aperture (NA) objective, with pinhole set to 1 Airy Unit (AU) using Zen Software (Carl Zeiss). Ventral Z-stacks (1-μm steps) were acquired, and analysis was performed using FIJI software. Intensity measurements were performed as described [63] with some modifications. Briefly, a region with an abundant neuroblast population (Hts^01103^), or individual neuroblasts (Hts^RNAi^), were selected using the polygon lasso tool in FIJI and Hts intensity was quantified using the Integrated Density (ID) function. Huygens Essential Deconvolution Suite (SVI) was used to deconvolve images and maximum intensity projections were generated. Figures were assembled in Adobe Illustrator.

### 4.5 Cloning and plasmid construction

cDNA cloning for bacterial protein expression was performed using PCR amplified fragments obtained from an S2 cell cDNA library template. Generation of 6×His fusion proteins (e.g., Mud^CC-PBD^, Pins^TPRs-Linker^, Insc^Asymm^, and various Hts constructs) utilized the pBH plasmid with 5′-*BamH*I or -*Bgl*II and 3′-*Xho*I or *Sal*I restriction sites. Generation of GST:Pins fusion utilized the pGEX plasmid with 5′-*Bgl*II and 3′-*Sal*I restriction sites. Generation of MBP:Hts fusions utilized the pMAL plasmid with 5′-*NdeI* and 3′-*SalI* restriction sites. Site-directed mutagenesis was carried out with a standard PCR protocol using KOD-XL DNA polymerase (EMD Millipore, catalog #71087).

### 4.6 Protein purification

All proteins were expressed in BL21 (DE3) *E. coli* under induction of isopropyl β-D-1-thiogalactopyranoside (IPTG) and grown in standard Luria–Bertani broth supplemented with 100 μg/mL ampicillin. Transformed cells were grown at 37°C to an OD_600_ ∼0.6 and induced with 0.2 mM IPTG overnight at 18°C. Cells were harvested by centrifugation (5,000 × *g* for 10 min), and bacterial pellets were resuspended in lysis buffer and flash-frozen in liquid nitrogen. Cells were lysed using a Branson digital sonifier and clarified by centrifugation (12,000 × g for 30 min).

For 6×His-tagged proteins, cells were lysed in N1 buffer (50 mM Tris pH8, 300 mM NaCl, 10 mM imidazole) and coupled to Ni-NTA resin (Thermo Fisher Scientific, catalog #88222) for 3 h at 4°C. Following extensive washing, proteins were eluted with N2 buffer (50 mM Tris pH8, 300 mM NaCl, 300 mM imidazole). The 6×His tag was removed using TEV protease during overnight dialysis into N1 buffer. Cleaved products were reverse affinity purified by a second incubation with Ni-NTA resin and collection of the unbound fraction. Final purification was carried out using an S200-sephadex size exclusion column equilibrated in storage buffer (20 mM Tris pH8, 200 mM NaCl, 2 mM DTT).

For GST- and MBP-tagged proteins used as bait in pulldown assays, cells were lysed in lysis buffer (50 mM Tris pH 8, 300 mM NaCl, 2 mM DTT), and lysate was then clarified by centrifugation (12,000 × g for 30 min) and immediately flash frozen in liquid nitrogen for storage at −80°C.

### 4.7 Pulldowns assays and interaction studies

Equivalent amounts of GST- or MBP-fused bait construct lysates (Pins or Hts) were absorbed to glutathione or amylose agarose, respectively, for 30 min at 4°C and washed three times to remove unbound protein. Subsequently, soluble prey proteins were added at varying concentrations for 2 h at 4°C with constant rocking in wash buffer (20 mM Tris, pH 8, 120 mM NaCl, 1 mM DTT, and 0.2% Triton-X100). Incubation for different times (e.g., 1 or 3 h at 4°C, or 1 h at room temperature) produced similar results, indicating that this experimental framework had established equilibrium binding conditions. Reactions were then washed four times in wash buffer, and resolved samples were analyzed by coomassie blue staining of SDS-PAGE gels. All gels shown in figures are representative of at least 3 independent experiments.

All interactions were quantified using ImageJ software. Briefly, gel images were converted to greyscale and individual band intensities were measured using the boxed “Measure” analysis tool. The size of measurement box was kept the same across all concentrations and was initially determined by the size of the largest bound band, typically at the highest concentration tested. To ensure accurate measurements of bound proteins, the intensities of bands for bound prey were normalized to that of the corresponding band for bait protein under each respective condition. Binding curves shown in figures plot these normalized intensities (expressed as arbitrary units, “AU”) as a function of prey protein concentration. Dissociation binding constants were calculated in GraphPad Prism using a one-site binding isotherm regression analysis. All plots and statistics were also performed in Prism.

### 4.8 Liquid-liquid phase separation assays

Proteins were combined at various concentrations in minimal buffer without addition of molecular crowding agents (20 mM Tris, pH 7.5; 120 mM NaCl) and equilibrated at room temperature for 5 min. Solutions were subsequently added to glass microscope slides in chambers assembled from affixed coverslip spacers. Coverslips were applied and samples were imaged at indicated time points for formation of LLPS droplets using a Nikon IX83 brightfield and fluorescence microscope.

## Data Availability

The original contributions presented in the study are included in the article/Supplementary Material, further inquiries can be directed to the corresponding author.
