# Supplementary material for: Drosophila Adducin facilitates phase separation and function of a conserved spindle orientation complex
Source: Front Cell Dev Biol. 2023 Aug 16;11:1220529. doi: 10.3389/fcell.2023.1220529 (PMC10467427; doi:10.3389/fcell.2023.1220529)
Supplement: Supplementary file 1 [file Table1.DOCX]

Supplementary Material

*Drosophila* Adducin facilitates phase separation and function of a conserved spindle orientation complex

Amalia S. Parra, Cameron A. Moezzi, and Christopher A. Johnston*

*** Correspondence:** Corresponding Author: [johnstca@unm.edu](mailto:johnstca@unm.edu)

# Supplementary Data

**Figure S1. Whole brain lobes from Control, *hts^RNAi^*, and *hts^01103^* show distribution of central brain Mir+ NBs.**

Representative images of larval CNS stained with Miranda (*top row, original image acquired at 488nm green; bottom row, greyscale converted*) for the indicated genotypes are shown. Brains are mounted ventral side up and images shown are maximal intensity projections of z-stack scans taken at 1 μm intervals. Brain lobes are indicated with dashed white circles. Yellow dashed lines indicate border between the central brain (*medial*) and optic lobe (*lateral*). Scale bar, 50 μm.

**Figure S2. Pins binds Insc *in vitro*.**

(A) GST alone (*lane 3*) or GST:Pins^TRPs^ (*lanes 4-10*) was immobilized on glutathione resin and incubated in the absence or presence of increasing concentrations of Insc^ASYMM^ (1-30μM; see *lane 1*). Whereas Insc bound GST:Pins^TPRs^ in a dose-dependent manner, no binding was noticeable with GST alone when incubated with the highest Insc concentration.

(B) Plot of saturation binding quantification demonstrating the affinity for the Insc interaction with GST:Pins^TPRs^. Curve shown was constructed using a one-site binding isotherm model, and the average ± standard deviation of the dissociation binding constants (K_D_) of 4 independent experiments is depicted in the box.

**Figure S3. Sequence analysis of Hts reveals presence of a C-terminal IDR.**

Domain diagram of Hts is depicted above a plot of PONDR scores as a function of Hts amino acid position (residue number). Scores exceeding 0.5 are considered to represent protein disorder. The Hts C-terminal tail is highly predicted to adopt an intrinsically disorder region (*dashed box*).
