# Supplementary figures and images for: Drosophila Adducin facilitates phase separation and function of a conserved spindle orientation complex
Source: Front Cell Dev Biol. 2023 Aug 16;11:1220529. doi: 10.3389/fcell.2023.1220529 (PMC10467427; doi:10.3389/fcell.2023.1220529)

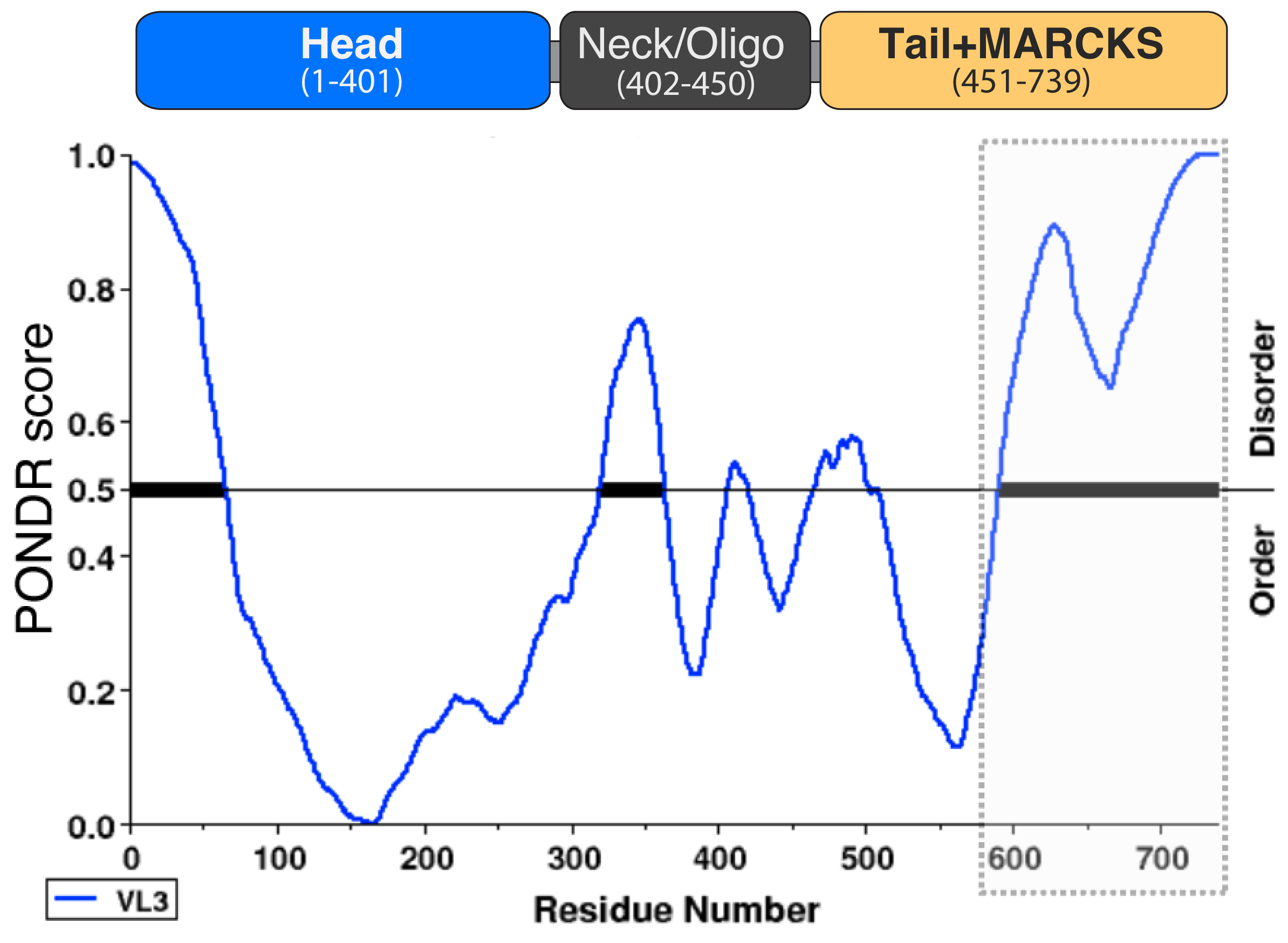

Supplement: Supplementary file 2 [file Image3.TIF]

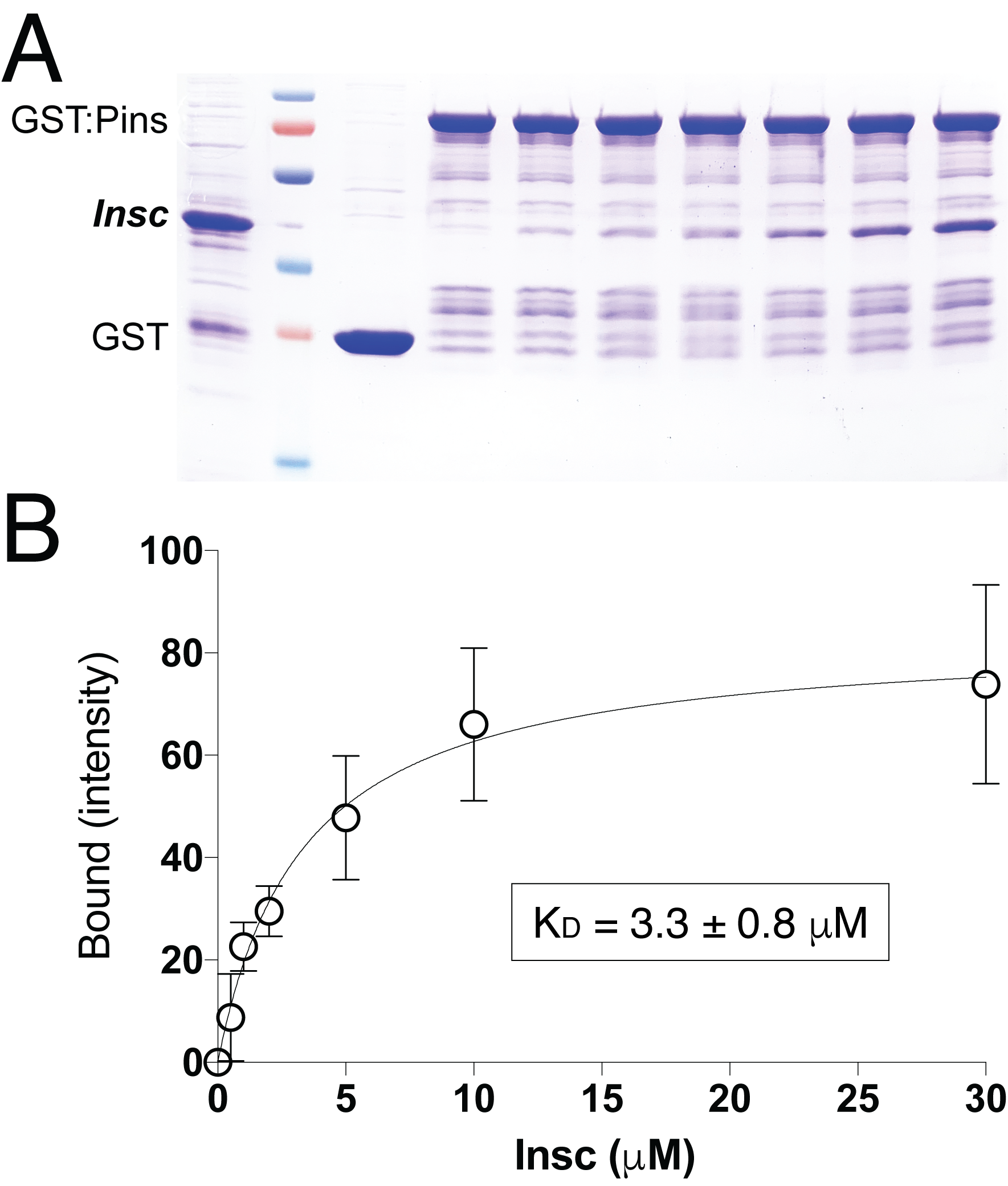

Supplement: Supplementary file 3 [file Image2.TIF]

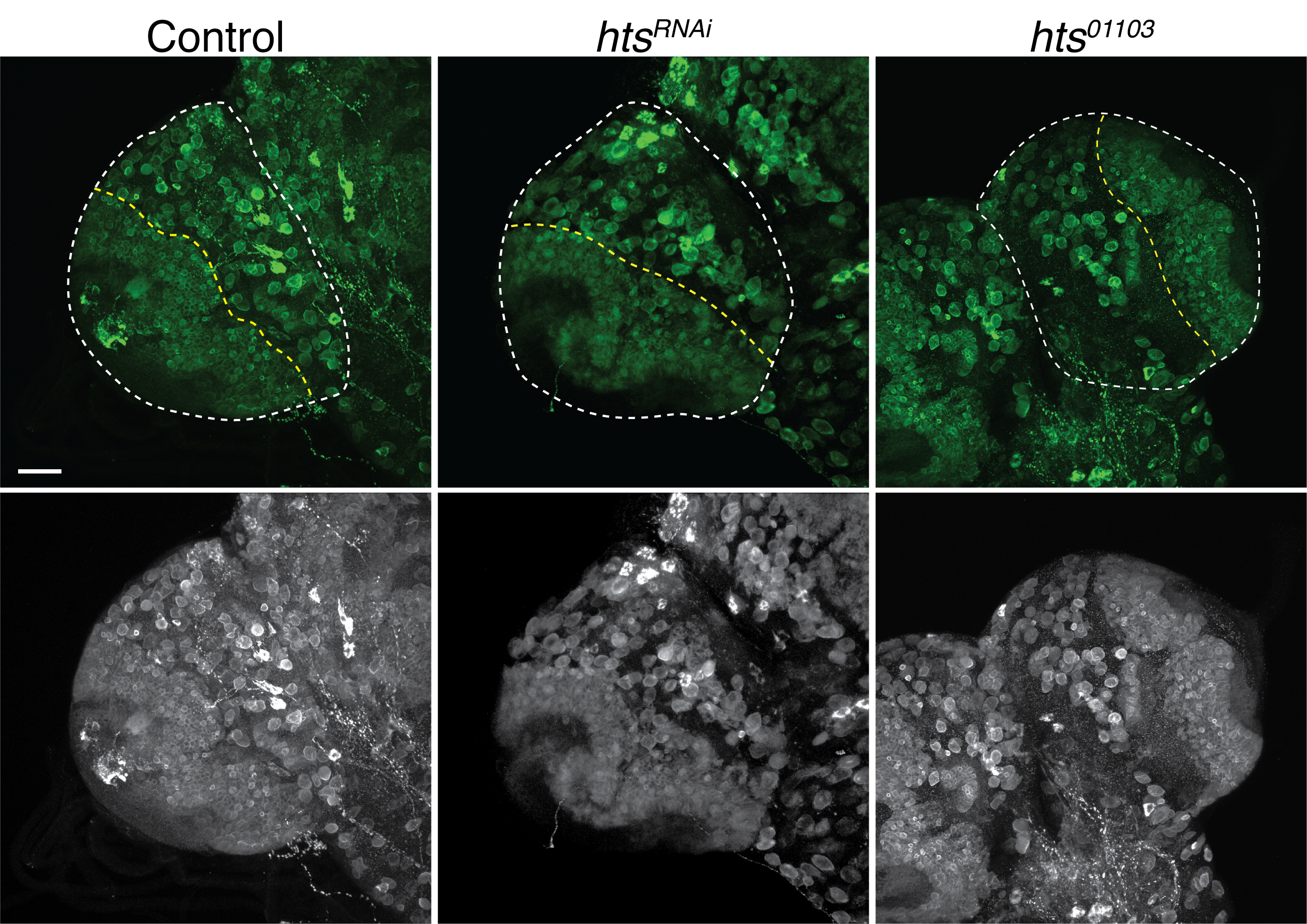

Supplement: Supplementary file 4 [file Image1.TIF]
